# Supplementary material for: Outcome of induction and associated factors among induced labours in public Hospitals of Harari Regional State, Eastern Ethiopia: A two years’ retrospective analysis
Source: PLoS One. 2021 Nov 9;16(11):e0259723. doi: 10.1371/journal.pone.0259723 (PMC8577748; doi:10.1371/journal.pone.0259723)
Supplement: S1 File — (PDF) [file pone.0259723.s001.pdf]

INDUCTION PROTOCOL (Ethiopian Federal Ministry of Health, 2010; WHO, 2011)

Induction of labor is the artificial stimulation of uterine contractions before the spontaneous onset of true labor at 28 or more weeks of gestation to achieve vaginal delivery

Induction methods

-S*urgical methods*: ARM, ballooned catheter, laminaria

-*Medical, pharmacological induction*: Oxytocin, prostaglandins such as misopristone

Indications

There are various indications. The criteria for termination of pregnancy and using induction (rather than CS) for each indication have to be referred to the specific condition dictating the induction.

The following list of indication is not exhaustive:

Preeclampsia, eclampsia, chronic hypertension, Diabetes mellitus, PROM, Chorioamnionitis, Abruptio placentae, Post-term pregnancy, Congenital abnormality, IUFD, Previous stillbirth, Rh isoimmunization,, IUGR

Contraindications

- ✓ *Contraindications for labor or vaginal delivery* such as placenta previa, transverse and oblique lie fetal lie, breech with contraindication for vaginal deliver (e.g., footling, extended neck). CPD, brow presentation, face with mento posterior, twin pregnancy, extensive genital wart, cervical cancer
- ✓ *Contraindications to oxytocin use* such as uterine scar (after CS, myomectomy, perforation, ruptured uterus etc), meconium stained amniotic fluid, NRFHP or fetal distress
- ✓ *Relative contraindications to oxytocin use* such as breech presentation (e.g., frank, complete), bad obstetric history, grand multi parity
- ✓ *Contraindication for ARM* such as cord presentations, placenta previa, vasa previa, active genital herpes with intact fetal membranes or less than 4 hours of rupture, presenting part above the pelvic inlet (relative)

Prerequisites

1. *Valid indication*: The indication for termination of the pregnancy entails that continuation with the pregnancy endangers the mother, fetus or both; and the benefits of pregnancy termination outweighs the benefits of continuing with pregnancy.

2. ***No contraindication***: There is not any foreseeable *contraindication* to achieve vaginal delivery (no indication for cesarean delivery at the time induction is initiated)

Elective induction: In case of an elective induction, lung maturity and Bishop Score are considered to minimize premature delivery and failed induction, respectively.

Assess fetal lung maturity: If the pregnancy is preterm or fetus does not have matured lung, consider using corticosteroid (GA<34 weeks) or allow pregnancy continue for some days if possible.

If lung maturity is ascertained, plan induction based on the Bishop score. The Bishop Score predicts *the likelihood of vaginal delivery* after induction with Oxytocin.

Bishop Scores

**Score**	**Dilation(cm)**	**Effacement (%)**	**Station**	**Consistency**	**Position**
0	Closed	0-30	-3	Firm	Posterior
1	1-2	40-50	-2	Medium	Mid-position
2	3-4	60-70	-1,0	Soft	Anterior
3	≥5	≥80	+1, +2	-	-

Interpretation of the Bishop's score:

Score<4: Unfavorable cervix is unlikely to yield for induction; cervical ripening is needed for success with induction. Postpone induction for next week if possible or use cervical ripening and plan induction for next day.

Score 5-8: Intermediate

Score = 9: Favorable cervical condition and induction is likely to succeed. There is no need for cervical ripening. Induction using Oxytocin can be planned for next day.

Methods of cervical ripening

Prostaglandin (PGE)

1. Intra-vaginal gel of 2.5mg PGE2 (Prostin) is applied on the upper vaginal canal (or posterior fornix) every 6 hours for a maximum of 4 doses.
2. Intra-cervical gel of 0.5 mg of PGE2 is placed in the endo-cervical canal and repeated after 6 hours for a maximum of 3 doses.
3. Vaginal insert of 3 mg PGE2 (Prostin), or 10 mg of dinaprostone is placed into the posterior fornix and repeated after 6 hours for a maximum of 4 doses.

4. Vaginal misoprostol of 25 mcg is placed into the upper vagina and repeated after 6 hours. If there is no response after 2 dozes of 25 mcg, the dose is increased to 50 microgram every 6 hours for a total of 200 mcg.

Schedule for escalating Oxytocin dosage

Dose and oxytocin concentration	Time	**Drops / minute**<br>1 ml ≈20 drops	**Approximate oxytocin in mIU/ minute**
First dose:<br>***2 IU of oxytocin in 1000 ml fluid***	0:00hrs<br>0:30hrs<br>1:00hrs<br>1:30hrs	20<br>40<br>60<br>80	2<br>4<br>6<br>8
Second dose:<br>***Add another 2 IU of oxytocin to the remaining first dose fluid***	2:00hrs<br>2:30hrs<br>3:00hrs	50<br>60<br>80	12<br>15<br>20
Third dose:<br>***Add another 2 IU of oxytocin on the remaining second dose fluid***	3:30hrs<br>4:00hrs<br>4:30hrs<br>5:00hrs<br>5:30hrs	50<br>60<br>80<br>As above<br>As above	24<br>30<br>40<br>As above<br>As above

Complications: Failure to initiate labor or achieve good contractions leading to failed induction leading to increased risk of cesarean section

- Atonic PPH
- Iatrogenic prematurity
- Uterine hyper stimulation/ tetanic contractions (oxytocin, PG)
    - Uterine rupture
    - Fetal distress
- Chorioamnionitis (prolonged rupture of membranes after ARM and repeated VE)
- Fetal sepsis and vertical HIV transmission (ARM)
- Cord prolapse (ARM)
- Placental abruption (ARM)
- Water intoxication (oxytocin)

- Amniotic fluid embolism

Failed induction

Definition: Failed induction is failure to initiate good uterine contraction. It is diagnosed if adequate uterine contractions are not achieved after 6 to 8 hours of oxytocin administration and use of the maximum dose for at least one hours.

Management

- If the induction is not for an emergency condition and the fetal membranes are intact (e.g. IUFD with unruptured membranes), the induction can be postponed and ripening of the cervix considered.
- If the pregnancy has to be terminated on the day of the induction or the membranes are ruptured, cesarean section is the only available option.
- Failed induction as an indication for cesarean section should be differentiated from other indications detected after achieving good uterine contraction. Protraction of labor after achieving adequate uterine contraction is managed as abnormal labor.

Differentiating CPD from failed induction as an indication for cesarean section is detrimental in planning the mode of delivery for subsequent pregnancies.

T*etanic contractions*

***Definition**:* Six or more contractions in 10 min and/ or durations of 90 or more seconds

Management

- Stop oxytocin infusion
- Use tocolytics if available
- Assess fetal and maternal conditions carefully for possible fetal distress or ruptured uterus. If there is fetal distress (e.g. NRFHP, meconium stained amniotic fluid) or uterine rupture, manage accordingly.
- If both mother and fetus are in good condition, restart at half dose of the last dose causing tetanic contractions.

References

1. FMOH 2010. MANAGEMENT PROTOCOL ON SELECTED OBSTETRICS TOPICS: Federal Democratic Republic of Ethiopia Ministry of Health.

2. WHO 2011. Recommendation to labor induction, Department of Reproductive Health and Research, World Health Organization, Avenue Appia 20, CH-1211 Geneva 27, Switzerland.

3. CUNNINGHAM, F., LEVENO, K., BLOOM, S., SPONG, C. Y. & DASHE, J. 2014. *Williams's obstetrics, 24e*, Mcgraw-hill
