# Supplementary material for: Outcome of induction and associated factors among induced labours in public Hospitals of Harari Regional State, Eastern Ethiopia: A two years’ retrospective analysis
Source: PLoS One. 2021 Nov 9;16(11):e0259723. doi: 10.1371/journal.pone.0259723 (PMC8577748; doi:10.1371/journal.pone.0259723)
Supplement: Attachment — Submitted filename: 3.Response to reviewers.docx [file pone.0259723.s003.docx]

**Authors’ Response to the editor’s and Reviewers’ comments and Suggestions**

**Manuscript ID:** PONE-D-21-06515

**Journal:** PLOS ONE

**Dear Editors and Reviewers,**

Thank you so much for giving us an opportunity to submit a revised draft of our manuscript entitled “***Outcome of Induction and Associated Factors among Induced Labors in Public Hospitals of Harar Town, Eastern Ethiopia: A two Years’ Retrospective Analysis”*** to this high visibility impact factor and peer reviewed Journal. We appreciate the time and effort that you and the reviewers dedicated to providing feedback on our manuscript. We are very grateful for the insightful comments and valuable improvements to our premature paper. We have incorporated most of the **suggestions** and **comments** made by handling editor, reviewers. All comments and suggestions are clearly stated and well addressed (a point-by-point to the reviewer’s comments and concerns). These changes are **highlighted in Red font color** within the clean revised manuscript.

**Authors’ Response to Editor’s Comments and Suggestions**

**Title*:*** *Outcome of Induction and Associated Factors among Induced Labors in Public Hospitals of Harari Regional State, Eastern Ethiopia: A two Years’ Retrospective Analysis*

**Authors:** Yimer Mohammed Beshir, Gudina Egata, Mohammed Abdurke Kure, Kedir Teji Roba,

**To: Handling Editor(s)**

**From: Mohammed Abdurke Kure (Corresponding Author)**

**Subject: Submission of *Incorporated Comments and Suggestions***

First, we thank you for your constructive comments and helpful suggestions that helped us to improve and enrich our manuscript. Here under in the table below, we have pointed out how authors incorporated your valuable comments, suggestions and concerns one by one.

| **Editor’s Comments to the Authors** | **Authors’ Responses to Editor’s Comments and Suggestions** |
| --- | --- |
| **Editor’s General Comments and Suggestions** | **Overall**, thank you so much for **cooperation** to handle our manuscript. Handling paper is really needs dedication and strong commitment. **Thanks a lot**! |
| **1**.Thank you for submitting your manuscript to PLOS ONE. After careful consideration, we feel that it has merit but does not fully meet PLOS ONE’s publication criteria as it currently stands. Therefore, we invite you to submit a revised version of the manuscript that addresses the points raised during the review process. | **Great!** We are very happy and overjoyed. Thank you very much for giving us an opportunity to submit our revised manuscript to such legitimate and high visibility impact factor Journal (PLOS ONE). |
| **Academic Editor’s Specific Comments (Journal Requirements)** |  |
| **2**. Please ensure that your manuscript meets PLOS ONE's style requirements, including those for file naming. The PLOS ONE style templates can be found athttps://journals.plos.org/plosone/s/file?id=wjVg/PLOSOne_formatting_sample_main_body.pdf and | **Thank you so much**. You are perfect**.** These are very important comments. Even it is the authors mandatory to stick to Journal’s format guidelines. Now, the authors addressed this critical issues based on your valuable suggestion. We downloaded all formats templates (PLOS Affiliations Formatting and Manuscript body formatting guidelines) from Journal’s Website, and critically read and corrected all necessary formatting. Newly changed and corrected were highlighted with red font color in the clean revised manuscript. |
| **3**. In the ethics statement in the manuscript and in the online submission form, please provide additional information about the patient records used in your retrospective study | **Thanks.** You are perfect**.** This valid comment was addressed based on your valuable suggestion. Newly changed and corrected was highlighted with red font color in the clean revised manuscript (on page, Lines= ) |
| **4.**Please remove any funding-related text from the manuscript and let us know how you would like to update your Funding Statement. | **Ok, thanks.** The editor is correct. We are very sorry; this is against PLOS ONE authors’ guideline. The authors critically reviewed this valid comment and corrected the necessary modification. The newly modified change was highlighted with red font color in the clean revised manuscript |
| **5.** If there are no restrictions, please upload the minimal anonymized data set necessary to replicate your study findings as either Supporting Information files. We will update your data availability statement on your behalf to reflect the information you provide. | **Thank you very much.** In fact, this is a valid concern. We critically considered this point, and we decided to upload data set used for analysis with revised submission as supplementary File 2(S2) |
| **6**. Your ethics statement should only appear in the Methods section of your manuscript. If your ethics statement is written in any section besides the Methods, please delete it from any other section. | **Thank you a lot for such a technical input**. Now, authors considered the raised issue. We are very sorry! In fact, this authors responsibility to follow and adhere Journal’s format guidelines. After thoroughly and critically revised this important comment, we removed from previously it appeared in Acknowledgment part. **Thanks!** |

**End of authors’ responses for Handling Editor(s)**

**Authors’ Response to Reviewer 1’s Comments and Suggestions**

Title***: Outcome of Induction and Associated Factors among Induced Labors in Public Hospitals of Harari Regional State, Eastern Ethiopia: A two Years’ Retrospective Analysis***

**Authors:** Yimer Mohammed Beshir, Gudina Egata, Mohammed Abdurke Kure, Kedir Teji Roba

**To: Reviewer 1**

**From:** Mohammed Abdurke Kure (Corresponding Author)

**Subject: Submission of *Incorporated Comments and Suggestions***

First and foremost, we would like to thank you for your constructive and valuable comments and helpful suggestions that helped us to improve and enrich our premature manuscript. Here under in the table below, we have pointed out how authors incorporated your valuable comments, suggestions and concerns one-by-one.

| **Reviewer’s Comments to the Authors** | **Authors’ Responses to Reviewer’s comments** |
| --- | --- |
| **Reviewer’s General Comments** | Overall, thank you very much for your positive and constructive suggestions |
| **1**. Is the manuscript technically sound, and do the data support the conclusions?  **Reviewer #1: partly** | **Thank you very much**. We thank you for your appreciation and constructive suggestion. Further, we revised and enriched the paper after previous initial submission to the journal. |
| **2.** Has the statistical analysis been performed appropriately and rigorously?  **Reviewer #1: No** | **Thank you so much**. Authors acknowledge you for your countless effort. We also appreciate this valuable response. Dear, reviewer, in this study, the authors followed standard methods of data collections and analysis. We used validated data collection tool, which was developed and customized from Ethiopian Demographic and Health Survey (EDHS) data collection tool, and by reviewing related published literature. We also used strong statistical software (Epi-Data version-3.1) for data entry. Further, data were exported to SPSS version 24 (IBM SPSS Statistics, 2016) for further analysis. Now, we revised the paper after initial submission. We took a long time, thoroughly revised and corrected the whole parts of our manuscript to make it more scientifically robust. Further, we also critically revised the statistical analysis of all descriptive statistics, Bi-variable and multivariable logistic regression models to correct any systematic errors introduced during analysis sage. |
| 3. Have the authors made all data underlying the findings in their manuscript fully available?  **Reviewer #1: Yes** | **Thanks a lot!** Authors acknowledged your countless efforts. Now, authors agreed to upload SPSS data set used for analysis as supplementary file 2(S2) with revised submission. |
| 4. Is the manuscript presented in an intelligible fashion and written in standard English?  **Reviewer #1: Yes** | **Thank you very much.** Even after initially submission, we thoroughly revised and edited the whole parts of our manuscript and extensively corrected all copy-editing errors in the clean revised manuscript. Authors also sent the manuscript to language expert/editor who critically reviewed, edited and corrected all language related errors made in a submitted manuscript. |
| **5. Specific Review’s Comments to the Authors** | **Authors Responses to Reviewers’ Specific comments** |
| **1.**The authors have conducted the research in an ethical fashion and collected all the data using appropriate mechanisms to ensure the quality remains appropriate for the subject. The authors have duly cited their peers and prior art and also conducted statistical analysis with rigour. However, the experimental setup does not indicate how this study is different from the prior studies and therefore provides no indication of why the outcomes might be expected to be different. If the setup was the same the output can be safely assumed to be the same. | **Thank you very much**. We would like to thank you for your appreciation and constructive suggestion. We really appreciate this valid observation. Based on your insightful suggestion, we took a long time and critical revised the paper to enrich it after previous initial submission to the journal. Dear, reviewer, in the introduction part, we clearly stated that no studies have conducted in the eastern Ethiopia, and even there were very few reports from other part of the country. Further, this study was conducted in eastern part of Ethiopia, particularly, Harari Regional State, where majority of the people are not accessed to health care services. This region has boundary with Somali Regional State, where marginalized and pastoralist community are inhibited, and many of laboring mothers referred to Hiwot Fana Specialized University Hospital for better care because it is the only Referral Hospital in eastern Ethiopia. |
| 2. Based on the current analysis shows no significant improvement in outcomes while utilizing IoL, further analysis is merited for the use case. | **Great! Thanks a lot.** Dear, reviewer, we really appreciate your valid concern. Initially, we followed standard methods of data collections and analysis. We also used strong statistical software (Epi-Data version-3.1) for data entry. Further, data were exported to SPSS version 24 (IBM SPSS Statistics, 2016) for further analysis. We used all statistical analysis steps required for comparison cross-sectional study (Descriptive statistics, Bi-variable analysis, Multivariable analysis). We performed regression analysis after critically considered assumption of logistic regression. Our outcome variable was “Binary outcome” and we dichotomized it accordingly. It is obvious that there would be limitation for “**cross-sectional study**” because no causal-association can be estimated. In this study, we used “**Facility-based Cross-sectional study** **with internal comparison**”, and we tried to increase our sample size (n=717) to have representative sample. Moreover, because of limited resource, we could not conduct, prospective longitudinal study to see the true causal-effect relationship (to estimate the true effect of predictor variables on the outcome of IOL). |
| **2a.** The paper seems to describe the effect of the parameters in isolation. The data representation, and therefore results, does not describe the effect of the combination of factors. Authors must conduct this analysis and clearly represent the significance of individual parameters and the various permutations and combinations | **Thank you so much**. Authors critically considered this input. Authors acknowledged your efforts. We critically considered and incorporated these raised issues. Dear reviewer, from the beginning, we followed all steps of statistical analysis as follows:  1.Questionnaire template was prepared using Epi-Dat version 3.1 for data entry  2.Double data entry was conducted by independent data clerk  3.After verification, entered data were exported to SPSS version 24 (**IBM SPSS Statistics, 2016**) for further analysis.  4. In SPSS analysis, imported data were cleaned and checked for outliers using (ascending and descending, simple frequency, Plot chat…etc).  5.Descript statistics were done using frequency tables, proportions and summary measures  6.Bivariable Analysis was conducted using Binary logistic regression analysis after dichotomizing of “outcome of induction as ‘Failed IOL’ and ‘Successful IOL’ ”. Thus, Failed IOL= 0 and Successful IOL= 1. Accordingly, around 12 predictors were run independently in respect with outcome of IOL.(**See Table 3)**  **7.** Multivariable analysis was performed after all assumptions of Logistic regression were fulfilled**.** Here, around 8 variables with p-value < 0.25 in Bivariable analysis were considered for multivariable analysis based on selection criteria(after model fitness and multicollineaity were checked**).(See Table 4)** |
| **2b.** A detailed description of the control sample (patients not receiving IOL) is required. The current manuscript does not provide details on this. | **Thanks a lot.** Authors appreciate your insightful concern. Dear, reviewer, in this study, we used the method of “Cross-sectional study design with internal comparison” which has been conducted in point in time or “**Snapshot**”. We have conducted retrospective chart-review of 2 years (**January 2017 to December 2018).** Moreover, in its nature, cross-section study does not have comparative group like case-control (Diseased Vs Non-diseased), Cohort study (Exposed Vs Unexposed group), RCT (Placebo Vs Interventional group). Therefore, In our case, we didn’t have control group, and our study subjects were “**Laboring mother who underwent IOL**”. Accordingly, the charts of mothers admitted to selected Public Hospitals in Eastern Ethiopia, who underwent labor induction(January 2017 to December 2018) were retrieved. |
| **2c**.Based on the results provided, the bishops score has a significantly higher effect on the outcome. While this may well turn out to be true, the analysis needs to be provide an analysis while holding the bishops score as a constant. This would allow a better study of the effect of each variable. Without this analysis the effect of the IOL on parameters beyond the bishops score are nebulous at best. A similar analysis should be conducted while holding each parameter constant. This would provide an exhaustive analysis that helps differentiate the output from prior work. | **Thank you a lot**. We appreciated your observation. We considered this deep and insightful concern. Dear, reviewer, initially, we followed all steps of statistical analysis. In the final model, multivariable analysis was performed after all assumptions of Logistic regression analysis were fulfilled**.** Accordingly, around **8 variables** with p-value<0.25 in Bi-variable analysis were considered for multivariable analysis based on selection criteria (after model fitness and multicollineaity were checked**).** These variables include (maternal age, residence, parity, Bishop score, PROM, NRFHBP, Methods of induction, Birth wt). In this final model analysis, all reference categories were held constant to estimate the effect of each predictor on outcome of IOL. In the previous submission, the Bishop score was also incorporated in both Bi-variable and multivariable analysis.  **(See Table 4)** |
| **3.** Authors should maintain unformity while describing results. Eg: Line 259 describes the paramenter as "more likely" based on the cut off; but the subsequent parameters are described as "less likely" at the cut off. The authors should construct the sentences to describe in one of two ways (either less or more). Switching between positive and negative effect of the cut off can create confusion for the readers. | **Thank you so much**. You are perfect. In fact, this is very important comment. This valid comment was addressed based on your insightful suggestion. Newly changed and corrected were highlighted with red font color in the clean revised manuscript (page , lines ). |
| 4. The discussion section focusses more on prior work than an analysis of the results. Perhaps correlation while holding parameters constant would provide more content for discussion. | **Great, Thanks a lot** for such implicit and critical review for our premature paper. You are perfect. We really appreciate this valid observation. Now, the authors critically reviewed this valid comment and corrected the necessary modification. The newly modified changes were highlighted with red font color in the clean revised manuscript |
| 5. Significant sections of the paper need to be re-written to correct for sentence construction. | **Thank you so much**. Authors critically considered this input. Authors acknowledged your efforts. We critically considered and incorporated all raised issues, comments, suggestions and concerns in this manuscript. Moreover, we thoroughly revised and edited the whole parts of our manuscript and extensively corrected all copy-editing errors in the clean revised manuscript. Authors also sent the manuscript to language expert/editor who critically reviewed, edited and corrected all language related errors made in a submitted manuscript. |
| 6. Suggestion: Some of the tables could be converted to graphical representation for easier interpretation. | **Ok, Great! Thanks a lot**. In fact, this is valid concern. It is obvious that graphs/bars are easier to capture the information about presenting data. In this study, we used 3 figures and 4 tables, and most Biomedical researchers recommend minimum number figures than tables. However, if is mandatory to put the data in the figure, we will modify based on your specific recommendation. **Thanks!** |
| **Overall the work conducted has potential for significant impact**, but a more detailed analysis is needed. In the current format the manuscript does not give any indication of differentiation from prior art. However, given the nature of the problem being addressed, I recommend the authors be give a chance to conduct a more details analysis and submit a renewed manuscript. | Thank you very much. Authors thank for your countless effort to enrich our paper. We also appreciate this valuable suggestion. Dear, reviewer, we tried to address your deep concern about the analysis of the data in the previous questions. Based on your insightful comments, the authors critically reviewed, and checked the SPSS archived data to correct any bolded errors or misleading results. However, the authors didn’t found any systematic errors that may introduced unintentionally. Moreover, we took a long time, critically revised the whole manuscript, and modified previously bulky paragraphs, poorly worded, and other copy-editing errors. All newly modified/changes were highlighted with red font color with in the revised main manuscript. For further details, we submitted SPSS data (used for analysis) to the Journal as supplementary file 2(S2) with the revised submission. |

### End of authors responses for Reviewer 1

**Authors’ Response to Reviewer 2’s Comments and Suggestions**

**Title*: Outcome of Induction and Associated Factors among Induced Labors in Public Hospitals of Harari Regional State, Eastern Ethiopia: A two Years’ Retrospective Analysis***

Authors: **Yimer Mohammed Beshir, Gudina Egata, Mohammed Abdurke Kure, Kedir Teji Roba**

**To: Reviewer 2**

**From:** Mohammed Abdurke Kure (Corresponding Author)

**Subject: Submission of *Incorporated Comments and Suggestions***

First and foremost, we would like to acknowledge you for your constructive and valuable comments and helpful suggestions that helped us to improve and enrich our manuscript. Here under in the table we have pointed out how authors incorporated your valuable comments, suggestions and concerns one by one.

| **Reviewer’s Comments to the Authors** | **Authors’ Responses to Reviewer’s comments** |
| --- | --- |
| **Reviewer’s General Comments** | **Overall,** thank you very much for your positive and constructive suggestions |
| **1**.Is the manuscript technically sound, and do the data support the conclusions?  **Reviewer #1: Yes** | **Thank you very much**! We appreciate your valuable response. |
| **2.** Has the statistical analysis been performed appropriately and rigorously?  **Reviewer #1: Yes** | **Thank you very much.** Authors acknowledged your countless efforts, positive and constructive suggestion! Further, we frequently revised the statistical analysis, and results to enrich the manuscript than previous its status. |
| 3. Have the authors made all data underlying the findings in their manuscript fully available?  **Reviewer #1: Yes** | **Thank you reviewer**! We are very grateful for your positive response. |
| 4. Is the manuscript presented in an intelligible fashion and written in standard English?  **Reviewer #1: Yes** | **Thanks a lot!** Authors also sent the manuscript to language expert/editor who critically reviewed, edited and corrected all language related errors made in a submitted manuscript. |
| **5. Specific Review’s Comments to the Authors** | **Authors Responses to Reviewers’ Specific comments** |
| 1 Abstract is written well and comprehensive | **Thank you very much**. We would like to thank you for your appreciation and constructive suggestion. Further, we revised and enriched the paper after previous initial submission to the journal. |
| 2. In the Introduction section, Authors may give more information about advantages and disadvantages of induction of labor (IOL) citing more literature | **Thank you a lot**. We accepted all your valid suggestion and concern in this paper, and correct accordingly. The newly modified part were highlighted with red-font color in the main manuscript. |
| 3. Study design, Study population, Eligibility criteria, and Sample size calculations are well planned and presented; sampling procedure is done well with good selection of variables. | **Thank you very much**. We would like to thank you for your appreciation and constructive suggestion. Further, we revised and enriched the paper after previous initial submission to the journal. |
| 4 The presentation of results obtained is very clear with facts of many pregnant women having indications of pre eclampsia/eclampsia and pre-labor rupture of membrane. The state of conditions are worrying in this part Ethiopia reported by the Authors. Parity of the mothers, nullipara and multipara mothers is also taken care in this study | **Thank you very much**. We thank you for your appreciation and constructive suggestion. Further, we revised and enriched the paper after previous initial submission to the journal. |
| 5. The results obtained are discussed well citing references. | **Ok! Thank you so much** |
| 6. As another parameter, in the discussion, the Authors would have touched upon socio-economic and nutritional status of pregnant women compared to those in other countries/Ethiopia. | **Thank you a lot**. We appreciated your observation. Now authors considered your valid concern and explicitly incorporated your input in the clean revised manuscript and highlighted with red font color. |
| 7. In this study Authors established that success of induction of labor was relatively low in study area | **Thank you very much**. We thank you for your appreciation and constructive suggestion. Further, we revised and enriched the paper after previous initial submission to the journal. |

**End of authors responses to Reviewer 2**
