# Supplementary material for: Outcome of induction and associated factors among induced labours in public Hospitals of Harari Regional State, Eastern Ethiopia: A two years’ retrospective analysis
Source: PLoS One. 2021 Nov 9;16(11):e0259723. doi: 10.1371/journal.pone.0259723 (PMC8577748; doi:10.1371/journal.pone.0259723)
Supplement: Attachment — Submitted filename: 3.Response to reviewers.docx [file pone.0259723.s004.docx]

**Authors’ Response to the editor’s and Reviewers’ comments and Suggestions**

**Manuscript ID:** PONE-D-21-06515

**Journal:** PLOS ONE

**Dear Editors and Reviewers,**

Thank you so much for giving us an opportunity to submit a revised draft of our manuscript entitled “***Outcome of Induction and Associated Factors among Induced Labors in Public Hospitals of Harar Town, Eastern Ethiopia: A two Years’ Retrospective Analysis”*** to this high visibility impact factor and peer reviewed Journal. We appreciate the time and effort that you and the reviewers dedicated to providing feedback on our manuscript. We are very grateful for the insightful comments and valuable improvements to our premature paper. We have incorporated most of the **suggestions** and **comments** made by handling editor, reviewers. All comments and suggestions are clearly stated and well addressed (a point-by-point to the reviewer’s comments and concerns). These changes are **highlighted in Red font color** within the clean revised manuscript.

**Authors’ Response to Editor’s Comments and Suggestions**

**Title*:*** *Outcome of Induction and Associated Factors among Induced Labors in Public Hospitals of Harari Regional State, Eastern Ethiopia: A two Years’ Retrospective Analysis*

**Authors:** Yimer Mohammed Beshir, Gudina Egata, Mohammed Abdurke Kure, Kedir Teji Roba,

**To: Handling Editor(s)**

**From: Mohammed Abdurke Kure (Corresponding Author)**

**Subject: Submission of *Incorporated Comments and Suggestions***

First, we thank you for your constructive comments and helpful suggestions that helped us to improve and enrich our manuscript. Here under in the table below, we have pointed out how authors incorporated your valuable comments, suggestions and concerns one by one.

| **Editor’s Comments to the Authors** | **Authors’ Responses to Editor’s Comments and Suggestions** |
| --- | --- |
| **Editor’s General Comments and Suggestions** | **Overall**, thank you so much for **cooperation** to handle our manuscript. Handling paper is really needs dedication and strong commitment. **Thanks a lot**! |
| **1**.Thank you for submitting your manuscript to PLOS ONE. After careful consideration, we feel that it has merit but does not fully meet PLOS ONE’s publication criteria as it currently stands. Therefore, we invite you to submit a revised version of the manuscript that addresses the points raised during the review process. | **Thank you so much.** We are very grateful for giving us an opportunity to submit our revised manuscript to such legitimate and high visibility impact factor Journal (PLOS ONE). Thank you editor. |
| **Academic Editor’s Specific Comments (Journal Requirements)** |  |
| **2**. Please review your reference list to ensure that it is complete and correct. If you have cited papers that have been retracted, please include the rationale for doing so in the manuscript text, or remove these references and replace them with relevant current references. Any changes to the reference list should be mentioned in the rebuttal letter that accompanies your revised manuscript. If you need to cite a retracted article, indicate the article’s retracted status in the References list and also include a citation and full reference for the retraction notice. | **Thank you so much**. You are perfect**.** These are very important suggestions. Even it is the authors mandatory to critically review references issue (Both in text-citation and Bibliography) to avoid any inconsistence and wrong citations across the document. Now, the authors addressed this critical issues based on your valuable suggestions. Newly changed and corrected References were highlighted with red font color in the clean revised manuscript. |

**End of authors’ responses for Handling Editor(s)**

**Authors’ Response to Reviewer 1’s Comments and Suggestions**

Title***: Outcome of Induction and Associated Factors among Induced Labors in Public Hospitals of Harari Regional State, Eastern Ethiopia: A two Years’ Retrospective Analysis***

**Authors:** Yimer Mohammed Beshir, Gudina Egata, Mohammed Abdurke Kure, Kedir Teji Roba

**To: Reviewer 1**

**From:** Mohammed Abdurke Kure (Corresponding Author)

**Subject: Submission of 3^rd^ Round *Incorporated Comments and Suggestions***

Above all, we would like to thank you for your constructive and valuable comments and helpful suggestions that helped us to improve and enrich our premature manuscript. Here under in the table below, we have pointed out how authors incorporated your valuable comments, suggestions and concerns one-by-one.

| **Reviewer’s Comments to the Authors** | **Authors’ Responses to Reviewer’s comments** |
| --- | --- |
| **Reviewer’s General Comments** | Overall, thank you very much for your positive and constructive suggestions |
| **1**. If the authors have adequately addressed your comments raised in a previous round of review and you feel that this manuscript is now acceptable for publication, you may indicate that here to bypass the “Comments to the Author” section, enter your conflict of interest statement in the “Confidential to Editor” section, and submit your "Accept" recommendation.  **Reviewer #1**: (No Response) | **Ok, great! Thank you so much**. We revised and enriched the paper after previous 2^nd^ round submission to the journal. Authors critically discussed on this reviewers suggestion (not endorsed for previous addressed comments) to the Journal’s query to enrich our paper to be scientifically sound, and we took a long time, implicitly revised for the reviewers concerns. Thus, now we clearly stated and addressed the this reviewer’s deep concern(particularly, the issue of novelty and duplication), in the question number 5 below(next page). **Thank you a lot, reviewer.** |
| 2. Is the manuscript technically sound, and do the data support the conclusions?  **Reviewer #**1: Yes | **Thank you.** Authors appreciate your positive response. |
| 3. Has the statistical analysis been performed appropriately and rigorously?  **Reviewer #**1: Yes | **Thank you so much**. Authors acknowledge you for your countless effort. We also appreciate this valuable response. |
| 4. Have the authors made all data underlying the findings in their manuscript fully available?  **Reviewer #**1: Yes | **Great!** Dear reviewer, we thank you again for constructive suggestion. |
| 5. Is the manuscript presented in an intelligible fashion and written in standard English?  **Reviewer #**1: Yes | **Thank you very much.** Even after revised 2^nd^ submission, we corrected all copy-editing errors in the clean revised manuscript after previous submission. |
| **5. Specific Review’s Comments to the Authors** | **Authors Responses to Reviewers’ Specific comments** |
| 1. Most comments have been addressed. However, the merit of conducting a study using identical parameters is debatable. I understand that the socio-economic and geographic settings of this study were different, but all previous studies on the same topic with varying socio-economic studies indicated the same output as the authors. This begs the question, is the study truly unique enough to add to a body of scientific knowledge. What was the scientific hypothesis to indicate that the outcome may be different in Ethiopia? What was the rationale for expecting a different outcome? This needs to be highlighted somewhere, else it is merely a repetition of the previous studies. | **Thank you very much**. We would like to thank you for your appreciation and constructive suggestion.  **Coming to the specific concern**: Really, this is an important intellectual suggestion. Dear, reviewer, authors really appreciate your implicit and deep concern about the novelty and repetition of this research paper. In this regard, we tried to indicate in the 2^nd^ round submission of the revised manuscript and review letter response.  Dear, reviewer, in the introduction part of this paper, we clearly stated that no studies have been conducted in the eastern part of Ethiopia, and even there were very few reports from other part of the country. Further, this study was conducted in eastern part of Ethiopia, particularly, Harari Regional State, where majority of the people are less accessed to health care services. Moreover, this Harari Reginal State has boundary with Somali Regional State, where marginalized and pastoralist community are inhibited, and many of laboring mothers referred to Hiwot Fana Specialized University Hospital for better care because it is the only Referral Hospital in eastern Ethiopia. Furthermore, in Ethiopia, eastern part of Ethiopia is one of the most neglected area of the country, particularly, Harari regional state which is located at periphery( at 526Km distance from capital Addis Aba) of the country, and has a boundary with Somali regional, where highly marginalized community(Pastoralists and semi pastoralist) communities are largely inhibited and seeking healthcare services from Harari regional state, Eastern Ethiopia.  - In Ethiopia, although few studies have been conducted in the last five years, almost all previous researchers were selective to central and Northern parts(Addis Ababa, Amhara, and Tigray regions), neglecting other regions of the country, particularly Afar, Harari, and Somali regions.  Further, the authors added researcher gaps that indicate the reasons why this study has been conducted in Eastern part of Ethiopia. The newly modified changes were highlighted within the clean revised manuscript on page (**page 5, lines 87-94).** **Thank you once again.**  **Dear,** reviewer, really publication is learning forum because we learned a lot from your insightful comments and suggestions throughout the review process our paper. |

### End of authors responses for Reviewer 1

**Authors’ Response to Reviewer 2’s Comments and Suggestions**

**Title*: Outcome of Induction and Associated Factors among Induced Labors in Public Hospitals of Harari Regional State, Eastern Ethiopia: A two Years’ Retrospective Analysis***

Authors: **Yimer Mohammed Beshir, Gudina Egata, Mohammed Abdurke Kure, Kedir Teji Roba**

**To: Reviewer 2**

**From:** Mohammed Abdurke Kure (Corresponding Author)

**Subject: Submission of *Incorporated Comments and Suggestions***

First and foremost, we would like to acknowledge you for your constructive and valuable comments and helpful suggestions that helped us to improve and enrich our manuscript. Here under in the table we have pointed out how authors incorporated your valuable comments, suggestions and concerns one by one.

| **Reviewer’s Comments to the Authors** | **Authors’ Responses to Reviewer’s comments** |
| --- | --- |
| **Reviewer’s General Comments** | **Overall,** thank you very much for your positive and constructive suggestions |
| 1.If the authors have adequately addressed your comments raised in a previous round of review and you feel that this manuscript is now acceptable for publication, you may indicate that here to bypass the “Comments to the Author” section, enter your conflict of interest statement in the “Confidential to Editor” section, and submit your "Accept" recommendation.  **Reviewer #2:** All comments have been addressed | **Great!** We are very grateful for your unquantified effort throughout the review process of our manuscript. Thank you so much. |
| **2**.Is the manuscript technically sound, and do the data support the conclusions?  **Reviewer #2: Yes** | **Thank you very much**! We appreciate your valuable response and constructive suggestion. |
| **3.** Has the statistical analysis been performed appropriately and rigorously?  **Reviewer #2: Yes** | **Thanks a lot.** Authors acknowledged your countless efforts, positive and constructive suggestion! |
| **4.** Have the authors made all data underlying the findings in their manuscript fully available?  **Reviewer #2: Yes** | **Thank you reviewer**! We are very grateful for your appreciation and constructive intellects. |
| **5**. Is the manuscript presented in an intelligible fashion and written in standard English?  **Reviewer #2: Yes** | **Thank you so much!** Authors also sent the manuscript to language expert/editor who critically reviewed, edited and corrected all language related errors made in a submitted manuscript. |
| **5. Specific Review’s Comments to the Authors** | **Authors Responses to Reviewers’ Specific comments** |
| **Reviewer #2**: Authors have corrected and modified the manuscript as per suggestions of reviewers, no more corrections | **Thank you very much**. We would like to thank you for your appreciation and constructive suggestion. Further, we revised and enriched the paper after previous 2^nd^ round submission to the PLOS ONE Journal.  **In conclusion**:  Really, this is leaning forum. Overall, we learned a lot from your comments and suggestion throughout the review process of this manuscript. **Thank you so much once again**. |

**End of authors responses to Reviewer 2**
